# Black-headed gulls synchronise their activity with their nearest neighbours

**DOI:** 10.1038/s41598-018-28378-x

**Published:** 2018-07-02

**Authors:** Madeleine H. R. Evans, Katie L. Lihou, Sean A. Rands

**Affiliations:** 1 School of Biological Sciences, University of Bristol, Life Sciences Building, Tyndall Avenue, Bristol, BS8 1TQ United Kingdom; 2 Bristol Veterinary School, University of Bristol, Langford House, Langford, North Somerset, BS40 5DU United Kingdom

## Abstract

Animals in groups can benefit from synchronising their behaviour, where multiple individuals conduct similar activities at the same moment in time. Previous studies have demonstrated that some species show synchronisation of vigilance behaviour, but have not explored the mechanism driving this behaviour. Synchronisation could be driven by animals copying their closest neighbours, which would mean that close proximity should lead to increased synchronisation. We simultaneously observed the behaviour of multiple individual black-headed gulls (*Chroicocephalus ridibundus*) within resting groups, and compared the activity of a focal individual with its two closest neighbours and a randomly selected control individual. Focal individuals were more likely to be synchronised with their closest neighbour. Synchronisation became less likely if individuals were not the closest neighbour. This suggests that synchronisation seen within groups is dependent upon the spatial positions of its members, and black-headed gulls pay more attention to their closest neighbours.

## Introduction

Spending time in groups gives animals many benefits^1^, such as enhancing foraging success^2,3^, or reducing predation risk^4–6^ or energetic expenditure^7^. Group members will show specific behaviours that enhance the advantages of being together, such as coordinating their activity to efficiently utilise a resource or achieve a goal. Mechanisms involving individuals paying attention to the actions of a small number of close individuals can make it possible to coordinate a larger group^8,9^. This is particularly apparent in large flocks of birds^10,11^ or shoals of fish^12,13^, which can self-organise if individuals within the group coordinate through paying attention to the actions of their nearest neighbours. Coordinated behaviour can also reduce the individual predation risk experienced by group members, when the vigilance behaviour of multiple individuals allows for the early detection of predators^5,14,15^. Although earlier theoretical predictions suggested that it is simplest for group members to scan for predators independently of each other^5,16^, recent work has shown that coordination of vigilance behaviour can sometimes be beneficial. This beneficial coordination can either have individuals spreading their vigilance behaviour so that they are not scanning for the predators at the same time as each other^17^, or synchronising their behaviour to scan at the same time^17,18^.

Some species of gull rest in loafing groups during the non-breeding season. In these groups, gulls show synchronisation of non-vigilant sleeping behaviour with their neighbours^19^, with focal individuals tending to be asleep when the nearest two neighbours are also sleeping. This behaviour leads to temporal waves of behaviour, where the group shows cyclical patterns of vigilance or sleep^20,21^. Although this temporal group synchronisation could be driven by individuals monitoring the behaviour of the entire group, it is perhaps more likely that they are paying more attention to their immediate neighbours, given that other group behaviours like flocking may only involve paying attention to the actions of close neighbours^10,11^. We would therefore expect to see a spatial relationship between individuals and their behaviour, with closer neighbours being more likely to be vigilant or non-vigilant at the same time^22^. Here, we test whether black-headed gulls (*Chroicocephalus ridibundus*) show this spatial synchronisation in their vigilance behaviour, by comparing the behaviours of individuals with neighbours and individuals that are further away.

## Materials and Methods

The Severn Estuary is home to a large wintering population of black-headed gulls^23^, consisting of both local breeders and winter migrants from the UK and northern Europe^24^. Between October and December 2017, we observed loafing groups of these gulls at a daytime aggregation site along the tidal River Avon, Bristol (looking at groups of birds collecting in the 2 km stretch between the Clifton Suspension Bridge and Gaol Ferry Bridge, with effort concentrated around the tidally-exposed mud banks around Brunel Way Bridge, 51° 26′ 55′′ N 2° 37′ 30′′ W). Observations were conducted within two hours of low tide. Most aggregations of birds seen (and all the groups considered here) consisted solely of black-headed gulls, although there were occasional visits from herring gulls (*Larus argentatus*), lesser black-backed gulls (*Larus fuscus*), and mallards (*Anas platyrhynchos*).

Groups of gulls were defined as all the birds associated no more than five body lengths away from each other, and we only considered groups that had a minimum of four individuals, and where there were no visual obstructions present^25^, such that all individuals had a clear line-of-sight to every other member of the group. The distance of five body-lengths was chosen following pilot testing, and was used because estimating individual separations beyond this length was logistically difficult for distant gulls. Choosing an arbitrary cut-off like this can have implications for generating larger association matrices^26^, but was sufficient for the simple relationships we consider here. Having identified a group, two observers waited at a maximum of 30 m from the birds for five minutes, to allow for habituation. At the end of this period, one individual in the group was randomly selected as a focal individual using the randomisation techniques described by Rands *et al*.^22^. After starting a timer, one observer recorded the behaviour of this focal gull after thirty seconds and then at thirty second intervals, collecting eleven consecutive observations. The gull was recorded as being vigilant if it had its head up and was moving it laterally. If its head was either down or facing forwards without moving, or its eyes were closed, or it was preening, we recorded its behaviour as non-vigilant. Wing and leg-stretches were also counted as non-vigilant behaviour. Each individual was observed for five seconds, and if it displayed any vigilance during this time, its behaviour was classified as vigilant for the period. At the same time as these observations, the other observer identified and recorded the behaviour of the closest and second-closest neighbour, along with a randomly selected control individual in the group that was neither the focal or one of its two closest neighbours. The distance of this random individual from the focal individual could therefore be anything between the distance from the focal bird to its third-closest neighbour and the distance from the focal to the furthest individual within the defined group. Because gulls could move during a series of observations, the identity of the neighbours and random gulls selected from observation to observation could potentially change, but the identity of the control bird was only changed if it either left the group or became one of the closer neighbours. After collecting a set of eleven datapoints on a group, the observers stopped recording and moved to identify a new group, and alternated tasks so that the observer following the focal individual changed. If the focal bird left the group, or if the group were disturbed and left before all eleven observations had been made, the observation attempt was abandoned. In total, 55 full sets of observations were made, and an additional 22 incomplete sets were abandoned for the reasons described (full data are available in Supplementary Data S1). The local population of gulls was estimated to be in the hundreds, and so we assumed that it was unlikely that we sampled previously observed focal individuals for each set of observations, minimising any effect of pseudoreplication through repeated sampling of the same individual.

Data were processed to calculate the proportion of observations that the nearest neighbour, second nearest neighbour, and control birds were conducting the same behaviour (using the ‘vigilant’ or ‘non-vigilant’ dichotomisation defined above) over the eleven observations. Because the focal individual was the unit of replication in this study, we compared synchrony proportions with a repeated-measures analysis of variance using a GLM in SPSS 23, after checking test requirements (the residuals were not skewed enough to impact on the results, and sphericity assumptions were met). *Post hoc* pairwise comparisons were conducted with a Bonferroni adjustment.

### Data availability

The dataset supporting this article is available as Supplementary Data S1.

### Ethical Statement

This observational study was carried out with the approval of the University of Bristol Animal Welfare and Ethics Review Body (UIN/17/064), and conducted in accordance with UK legislation.

## Results

**Figure 1:**
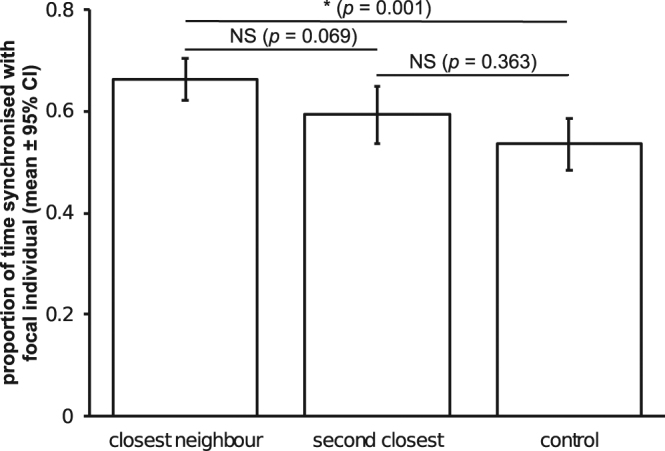
Mean proportion of time that the focal bird was synchronised with individuals of differing social proximity to it. *Post hoc* pairwise comparisons are reported above the bars.

Gulls differed in their behavioural synchrony dependent upon proximity to the focal bird (*F*_2,108_ = 7.334, *p* = 0.001, Fig. 1), where the focal bird was vigilant for 37.4 ± 26.7% of observations (mean ± SD). The amount of synchronisation fell as the social proximity to the focal individual was reduced, with the randomly picked control birds being much less synchronised than the closest neighbour to the focal (*p* = 0.001, Fig. 1).

## Discussion

This study demonstrates that resting black-headed gulls tend to synchronise their vigilance behaviour with conspecifics who are close to them. This synchronisation is strongest with the closest neighbour, and there is a trend towards synchronisation reducing as the topological distance between individuals increases (Fig. 1), echoing similar patterns seen in red deer (*Cervus elaphus*)^22^ and in cows (*Bos taurus*)^27^. This means that as well as potentially showing temporal waves of synchronisation (as seen in other species of gull^20^), gulls may also show spatial patterns of synchronised behaviour.

More work would be required to identify the mechanism for the synchronisation that we observed. Synchrony within groups can emerge through social facilitation^28–30^, where an individual is more likely to conduct a behaviour when in the presence of another individual that is already engaged in conducting the behaviour. Alternatively, synchrony can result from some external local influence or *zeitgeber*^31,32^ causing similar responses to occur in multiple individuals at the same time. Studies that have suggested that synchronisation is induced by local stimuli usually consider behaviours that require individuals to be aggregated at a local resource, such as foraging behaviour seen at a feeder (*e*.*g*.^32–34^). The loafing behaviour we recorded here is less likely to be either clustered or influenced by local stimuli than feeding behaviours, but could conceivably be driven by local habitat differences in predation risk that affect vigilance behaviour, such as exposure and proximity to cover^35–39^, as well as position within the group and proximity to other individuals^4,6,40–43^. Both the influence of other individuals or environmental factors could have caused the synchronisation we observed here. We could attempt to pick apart these mechanisms by tracking the spread of a behaviour within the group using continuous sampling techniques, and we could record the spatial position and orientation of individuals along with their proximity of potentially influencing environmental factors. However, without intentional experimental manipulation, it is unclear whether there are predictable differences that separate the possible mechanisms causing the synchronisation behaviour that we observed, and we call for more sophisticated experiments and analytical techniques (such as those described in^44–49^) that would allow us to tease apart the mechanisms causing spatial synchronisation in groups. Any experimental manipulation of either wild resting groups or their environment is not a trivial task: the effects of social composition could be investigated using managed groups where individuals are identifiable and can be removed, whilst the effects of perceived predation risk and disturbance could be investigated by manipulating cover or cues that indicate predator presence.

We assume that individuals are simply synchronising their behaviour with whichever individual is closest, but other factors may be important for driving how individuals interact with each other. As well as the physical constraints experienced by the gulls in monitoring each other such as orientation^50^ and the visual field^51^ and the exact behavioural rules they are following^52^, individuals may mediate their behaviour dependent upon their sex, age or social status^53^, social affiliation^54^, energetic state^55,56^, personality^57^ or some other benefit to be gained from synchronising with a neighbour’s behaviour^58^. Both modelling^59^ and empirical evidence^17,19,60^ also suggest that the likelihood of synchronisation occurring will be influenced by group size, distance between individuals, and the level of disturbance and predation risk, where these different factors are likely to interact in a complex manner^61,62^. We also only took point-samples of behaviour at defined moments in time, and may have missed subtler, fast behaviours, such as briefly scanning to check on the behaviour of their neighbours^19^.

Although much work has looked at how individual behaviours can lead to complex group behaviours, surprisingly little has been done to explore how behaviours can spread within groups of animals that are known to synchronise with each other^22^. Using a simple observational technique like the one discussed here can allow us to explore how proximity affects behaviour, and could easily be applied to other species that collect in observable static groups. Copying the vigilance of neighbours could lead to spatial waves of vigilance in groups, and there is scope for more sophisticated theoretical work and observations that allow us to explore how collective behaviours start and spread within groups.

## Electronic supplementary material

Dataset 1
